# An Analysis of a Transposable Element Expression Atlas during 27 Developmental Stages in Porcine Skeletal Muscle: Unveiling Molecular Insights into Pork Production Traits

**DOI:** 10.3390/ani13223581

**Published:** 2023-11-20

**Authors:** Chao Wang, Bowen Lei, Yuwen Liu

**Affiliations:** 1 Key Lab of Agricultural Animal Genetics, Breeding and Reproduction of Ministry of Education and Key Laboratory of Swine Genetics and Breeding of Ministry of Agriculture, College of Animal Science and Technology, Huazhong Agricultural University, Wuhan 430070, China; wangchao2020@webmail.hzau.edu.cn (C.W.); leibowen@webmail.hzau.edu.cn (B.L.); 2 Shenzhen Branch, Guangdong Laboratory for Lingnan Modern Agriculture, Key Laboratory of Livestock and Poultry Multi-Omics of MARA, Agricultural Genomics Institute at Shenzhen, Chinese Academy of Agricultural Sciences, Shenzhen 518124, China; 3 Innovation Group of Pig Genome Design and Breeding, Research Centre for Animal Genome, Agricultural Genomics Institute at Shenzhen, Chinese Academy of Agricultural Sciences, Shenzhen 518124, China; 4 Kunpeng Institute of Modern Agriculture at Foshan, Chinese Academy of Agricultural Sciences, Foshan 528226, China

**Keywords:** TEs, WGCNA, pig, development stage, gene regulatory network

## Abstract

**Simple Summary:**

The quality and yield of pork are significantly affected by the development and growth of porcine skeletal muscle. To identify the genetic factors that underlie this biological process, we initiated a study focusing on transposable elements (TEs). Due to their capacity of self-replication and relocation within the host genome, TEs play an important role in shaping the genome regulatory landscape. By utilizing extensive transcriptomic data spanning 27 developmental stages of porcine skeletal muscle, we created a comprehensive atlas of TE expression, revealing the intricate relationship between active TEs and epigenetic modifications. Through gene co-expression network analysis and the integration of multi-omics data, we uncovered a TE-mediated genetic regulatory network governing the development and growth of skeletal muscle. This research not only identifies valuable candidate genes for the genetic improvement of the economic traits of pork but also introduces an innovative TE-centric approach for dissecting the genetic factors contributing to complex traits.

**Abstract:**

The development and growth of porcine skeletal muscle determine pork quality and yield. While genetic regulation of porcine skeletal muscle development has been extensively studied using various omics data, the role of transposable elements (TEs) in this context has been less explored. To bridge this gap, we constructed a comprehensive atlas of TE expression throughout the developmental stages of porcine skeletal muscle. This was achieved by integrating porcine TE genomic coordinates with whole-transcriptome RNA-Seq data from 27 developmental stages. We discovered that in pig skeletal muscle, active Tes are closely associated with active epigenomic marks, including low levels of DNA methylation, high levels of chromatin accessibility, and active histone modifications. Moreover, these TEs include 6074 self-expressed TEs that are significantly enriched in terms of muscle cell development and myofibril assembly. Using the TE expression data, we conducted a weighted gene co-expression network analysis (WGCNA) and identified a module that is significantly associated with muscle tissue development as well as genome-wide association studies (GWAS) of the signals of pig meat and carcass traits. Within this module, we constructed a TE-mediated gene regulatory network by adopting a unique multi-omics integration approach. This network highlighted several established candidate genes associated with muscle-relevant traits, including HES6, CHRNG, ACTC1, CHRND, MAMSTR, and PER2, as well as novel genes like ENSSSCG00000005518, ENSSSCG00000033601, and PIEZO2. These novel genes hold promise for regulating muscle-related traits in pigs. In summary, our research not only enhances the TE-centered dissection of the genetic basis underlying pork production traits, but also offers a general approach for constructing TE-mediated regulatory networks to study complex traits or diseases.

## 1. Background

The pig holds a crucial place in agriculture, serving as a primary protein source for humans. Over a long period of geographical isolation and differences in domestication selection, Eastern and Western pig populations have developed significant variations in various economically important traits. For example, Western pigs typically exhibit higher growth rates and superior meat production performance, while Eastern pigs show a stronger tolerance to roughage, slower growth rates, and a higher fat content [1,2]. Prior research has highlighted the disparities in the genome-wide genetic variation landscape [3,4] and gene expression profiles across various tissues [5] between Western and Eastern pig breeds. Enhancing pork production performance has been a longstanding goal for breeders and farming enterprises. Duroc, Landrace, and Large White pigs represent prominent Western pig breeds, well-known for their outstanding pork production capabilities. These breeds are commonly utilized as breeding stock in swine production and serve as invaluable genetic resources for investigating the genetic factors influencing meat production.

It is well established that the development and growth of skeletal muscle, which comprises a significant portion of a pig’s body, are closely linked to economic traits that are relevant to meat production. Therefore, since the advent of genetic breeding, deciphering the genetic regulatory mechanisms governing porcine skeletal muscle development and growth has been of significant importance in accelerating the improvement of pork production traits. In the past decade, the application of GWAS analysis has led to the identification of numerous single nucleotide polymorphisms (SNPs) that are associated with pig meat production traits. For instance, Zhou and colleagues conducted a meta-analysis of GWAS in Duroc pig populations, pinpointing SNPs that are associated with average daily gain (ADG) and lean meat percentage (LMP) [6]. Gao and collaborators, in a three-way crossbred commercial pig population, discovered SNPs that are significantly associated with traits such as conductivity, intramuscular fat (IMF), marbling score, meat color, moisture, and pH [7]. Additionally, several genes influencing pork meat production traits have been reported, including the Hal and RN genes [8], IGF2 [9], PHKG1 [10], and MC4R [11]. The discovery of these SNPs and genes has provided crucial support for enhancing the genetic improvement in meat production traits.

Skeletal muscle development and growth consist of two sequential phases: myofiber formation before birth and myofiber hypertrophy after birth [12,13]. The initial phase spans the embryonic and fetal stages, crucial for the maturation of primary and secondary myofibers and the establishment of the muscle fiber count. The subsequent neonatal period constitutes the second phase, marked by a transition from slow to fast myofiber types, significantly influencing pork quality [14]. The adult phase, within the second phase, aligns with the growth and fattening stage of muscle development [15]. These distinct temporal effects on pork production highlight the dynamic regulatory networks that govern skeletal muscle growth and development, emphasizing the need for comprehensive investigations spanning various developmental time points. To meet this need, recent breakthroughs in multi-omics technology have been extensively exploited in pig samples. Studies have examined the dynamic alterations in DNA methylation patterns [16,17], fluctuations in mRNA or noncoding RNA expression profiles [18,19,20], dynamic changes in chromatin accessibility [21,22], alterations in the three-dimensional structure of the genome [23], and changes in the histone modifications associated with diverse cis-regulatory elements [24]. Undoubtedly, they have markedly advanced our comprehension of the genetic regulatory mechanisms governing the development of pig skeletal muscles. However, there is a category of regulatory elements that is frequently underestimated, constituting approximately half of the mammalian genome—TEs.

TEs are repetitive DNA sequences that are capable of replicating and relocating within a host genome. With the advancements in sequencing technologies, research on TEs has broadened, revealing that their distribution in the genome is far from random [25,26]. It is widely acknowledged that TEs play vital functional roles in the genome. They act as a significant source of mutations and genetic polymorphisms [27], orchestrate genome rearrangements [28], and modulate gene expression by forming cis-regulatory elements [29,30]. Additionally, they facilitate the generation of both coding [31] and noncoding RNAs [32]. Among these biological processes, TE transcription is deemed to be of considerable importance but remains relatively understudied. While many TEs have lost their ability to generate new insertions over their evolutionary history, they can still be transcribed from their genomic positions. To deepen our comprehension of the significance of TE transcription, a variety of bioinformatics tools have been developed in recent years to quantify TE transcription levels. Notable examples of these software tools include SQuIRE (v0.9.9.9a-beta), Telescope (v1.0.3) [33], TEtranscripts (v2.2.3) [34], scTE (v1.0.0) [35], and SoloTE (v1.09) [36]. These advancements have paved the way for novel investigations into how TE expression might affect complex traits.

In this study, we employed a widely utilized TE quantification tool, SQuIRE [37], to accurately measure TE expression levels in Landrace porcine skeletal muscle tissue across 27 developmental stages. The extensive timepoint coverage within this TE atlas facilitates a more robust construction of co-expression-based regulatory networks, enabling the subsequent identification of pivotal TE-mediated regulatory modules and potential target genes that likely play a significant role in governing skeletal muscle development. Our study not only sheds light on how TEs might impact pork production traits but also provides a novel analytic framework for evaluating the genetic contribution of TEs in other economically important traits of farm animals.

## 2. Materials and Methods

### 2.1. RNA-Seq Data Analysis

The RNA-seq data for Landrace skeletal muscle (longissimus dorsi) used in this study were sourced from our prior publication (SRP158448) [38], with three biological replicates for each developmental stage. These comprehensive transcriptomic datasets were collected across 27 distinct developmental phases in Landrace pigs. This included embryonic stages spanning days 33, 40, 45, 50, 55, 60, 65, 70, 75, 80, 85, 90, 95, 100, and 105, conveniently abbreviated as LE33, LE40, LE45, LE50, LE55, LE60, LE65, LE70, LE75, LE80, LE85, LE90, LE95, LE100, and LE105. In addition, the postnatal stages encompassed days 0, 9, 20, 30, 40, 60, 80, 100, 120, 140, 160, and 180, abbreviated as LD0, LD9, LD20, LD30, LD40, LD60, LD80, LD100, LD120, LD140, LD160, and LD180. This dataset forms the basis of our investigation. Raw reads of RNA-seq were filtered using Trim Galore and were subsequently aligned to the pig genome using Hisat2 [39] (v2.2.1, --rna-strandness RF). The featureCounts tool [40] (v2.0.1) was employed to quantify the reads assigned to each gene, based on the genomic coordinates of gene exons listed in the gene annotation file. To normalize gene expression levels, the TPM (transcripts per million) values were calculated, and further adjustments were made for the library size using edgeR [41]. This process resulted in the creation of a final gene expression matrix that encompasses all developmental stages.

### 2.2. TE Annotation and Quantification

We downloaded the reference genome sequence for pigs (susScr11.fa) from the UCSC database [42] for TE annotation. The TE annotation process was carried out using RepeatMasker software (v4.1.2-p1) [43] with the Dfam_Consensus-20181026 and Repbase-20181026 libraries (-species pig -html -s -a -gff -e rmblast -gccalc -no_is -dir susScr11 susScr11.fa). After obtaining TE location annotation information for the pig genome, we utilized the high-performance SQuIRE software (v0.9.9.9a-beta) [37] to perform TE expression quantification by integrating BAM files from the 27 different stages of transcriptomes with the default parameters. Ultimately, TEs with an average expression level (fragments per kilobase million, FPKM) exceeding 1 across the three replicate samples were categorized as expressed TEs for that particular developmental stage. Nonexpressed TEs at that stage were defined as those lacking sufficient transcription signals.

### 2.3. Multivariate Analysis of Detectably Expressed TEs

A dynamic heatmap of expressed TEs across the 27 examined developmental stages was generated using the R package Complexheatmap (v2.6.2) [44]. The principal component analysis (PCA) clustering analysis was conducted on samples from all 27 stages at both the gene and TE levels using the R program’s “prcomp” function. The first two principal component dimensions were utilized to generate graphical visualizations. The genomic distribution annotation of TEs was performed using the R package ChIPseeker (v1.26.2) [45]. To investigate the relationship between the expression levels of TEs and their methylation levels, we acquired a matching methylation dataset for Landrace skeletal muscle across the 27 developmental stages, aligning precisely with the samples in our current RNA-seq analysis. This dataset was sourced from GSE157045, providing a seamless fit for our ongoing study [16]. To assess the degree of methylation at each CpG site, we calculated the proportion of methylated reads relative to the total reads. For each genomic region, we determined the average methylation level by calculating the ratio of methylated reads across all internal CpG sites relative to the total reads. Finally, TEs identified at each stage with observable expression levels were ranked based on their expression levels and classified into three groups: “low” denotes TEs expressing within the 0–0.3 percentile, “medium” encompasses TEs within the 0.3–0.6 percentile, and “high” refers to TEs expressing above the 0.6 percentile. Subsequently, we conducted a comparison of the methylation levels among these TEs within each of the three categories. To explore the differences in chromatin accessibility and active histone modifications between transcribed and nontranscribed TEs, we collected publicly available signal peak position information for ATAC, H3K27ac, and H3K4me3 in pigs [46]. We conducted permutation tests by simulating TEs in the genome 1000 times to assess whether TEs are enriched in these epigenetic regulatory regions. In addition, in our study, the gene ontology (GO) analysis of TEs was conducted using ChIPseeker to extract the nearest genes to TEs based on their proximity to gene transcription start sites (TSS). Subsequently, the analysis of GO term enrichment was carried out using the clusterProfiler software (v3.18.1) [47], which is based on the principles of hypergeometric distribution.

### 2.4. Weighted Gene Co-Expression Analysis

We performed weighted gene co-expression analysis on the expression matrix of 6074 self-expressed TEs across the 27 examined stages of skeletal muscle development using the R package WGCNA (v1.69) to uncover their key modules in muscle development. The analysis comprised the following main steps: Firstly, we used the pickSoftThreshold function to determine the best soft threshold (β value) for constructing the gene co-expression network. Next, we divided genes into different co-expression modules using the topological overlap matrix (TOM) dissimilarity as the clustering distance measure to capture co-expression relationships between genes. To better understand the organizational structure of the modules, we utilized the plotDendroAndColors function to generate a module clustering dendrogram and assigned different color labels to different modules to visually display their distribution and composition. Finally, we determined the final modules using the blockwiseModules function with the parameters minModuleSize = 30, TOMType = “unsigned”, pamRespectsDendro = FALSE, and mergeCutHeight = 0.25. To explore the correlation between the modules and the regulation of muscle development growth stages, following previous research, we divided muscle development into four states based on the physiological characteristics of skeletal muscle at different stages: primary myofiber formation, second myofiber formation, myofiber type transition, and the fattening phase. We calculated the correlation between different modules and these states using the cor function.

### 2.5. Module Function Analysis

A functional enrichment analysis of the modules was conducted by extracting nearby genes of TEs within the modules using the clusterProfiler package. The GWAS data for the pig were analyzed—related traits used in this study were collected from the animal QTLdb database [48]. Within this database, pig traits are classified into five distinct categories: Meat and Carcass, Health, Production, Reproduction, and Exterior. To assess TE trait associations, we performed an enrichment analysis by conducting 1000 permutations, randomizing the distribution of TEs across the genome. It is important to note that, considering the linkage disequilibrium effects between SNPs, GWAS-associated variant loci were extended by ±20 kb. In addition, motif enrichment analysis of the TEs was performed using Homer software (v4.11) [49].

### 2.6. TE-Mediated Gene Regulatory Network Construction

The identification of TE target genes was conducted by utilizing the bedtools’ closest plugin to extract genes located upstream and downstream of TEs. Subsequently, we analyzed the correlation between TE and adjacent gene expression levels across 27 developmental stages using the psych package’s corr.test function (v2.2.5) with the parameter method = “spearman” and adjust = “fdr”. Genes with an adjusted *p*-value (p.adj) of <0.05 and a correlation coefficient (Rs) of ≥0.3 were defined as being positively regulated by TE, while genes with p.adj < 0.05 and Rs < −0.3 were defined as being negatively regulated by TE. Through this coordinated change in correlation, we identified TE–gene pairs. Furthermore, we employed the exportNetworkToCytoscape() function from the WGCNA package, combined with a threshold of 0.2, to filter reliable TE–TE interaction pairs within modules, thereby creating an initial TE–TE–gene regulatory network. Finally, by overlapping Meat and Carcass GWAS-associated loci extended by 20 kb with TEs, we filtered out regulatory relationships between TE and GWAS loci, resulting in the formation of the ultimate hub TE–TE–gene regulatory network governing muscle development. Visualization of the hub TE–TE–gene regulatory network was performed using Cytoscape software (v3.10.1) [50], and node importance scores were color-mapped using the NCC algorithm from the cytoHubba plugin (v0.1) [51].

## 3. Results

### 3.1. The Construction of a Dynamic Expression Atlas of TEs

In our study, we examined the expression profiles of TEs in porcine skeletal muscles across 27 distinct developmental phases, spanning from embryonic to adult stages, by using SQuIRE software (v0.9.9.9a-beta). To our knowledge, this marks the creation of the first-ever dynamic expression atlas illustrating the behavior of TEs during porcine muscle development. Notably, this atlas is the most comprehensive TE expression atlas, offering a more thorough coverage of developmental timepoints than any other TE expression atlas across all known species.

**Figure 1:**
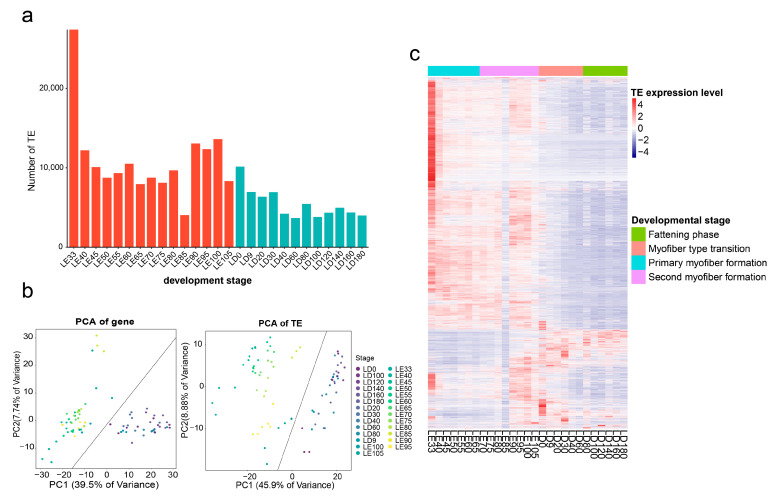
Dynamic expression patterns of TEs across different developmental stages in skeletal muscle. (**a**) Quantification of TEs with detectable transcription at various developmental stages. Red bars indicate pre-birth stages, while blue bars denote post-birth stages. (**b**) PCA depicting the similarity in gene and TE expression patterns across all samples. (**c**) Heatmap illustrating the expression signals of all detectable TEs at different developmental stages.

Across these 27 different developmental stages, we successfully identified a total of 39,640 expressed TEs. Notably, the number of expressed TEs in porcine muscle tissue during the embryonic stage was higher than that observed after birth (Figure 1a). Among these developmental stages, LE33 exhibited a notably high abundance of expressed TEs. We conducted a GO analysis of the specifically expressed TEs in LE33 and discovered their predominant involvement in fundamental biological pathways, such as mRNA metabolic processes, BMP signaling pathways, and Wnt signaling pathways (Supplementary Figure S1). This suggests that TEs play a crucial role in maintaining essential metabolic processes during the early stages of porcine skeletal muscle tissue development.

To investigate the similarity of TE and gene expression patterns across different developmental stages of skeletal muscle, we conducted sample PCA clustering analyses across the 27 time points. Two PCA plots were generated, one based on the expressed genes and the other on TEs (Figure 1b). We found that, in both cases, the first two principal components (PCs) explained a substantial proportion of the gene expression variation across skeletal muscle development. In these two PCs, gene expression accounted for 47.2% of the total variance, while TE expression demonstrated an even greater explanatory power, covering 54.78% of the overall variance. Furthermore, it is worth noting that the first two PCs effectively distinguished between prebirth and postbirth conditions, suggesting that changes in TE expression dynamics can accurately reflect the characteristics associated with different developmental stages. Based on the physiological characteristics of porcine skeletal muscle development, we further subdivided the entire developmental process into four distinct periods: primary myofiber formation (LE33 to LE65), secondary myofiber formation (LE70 to LE105), myofiber type transformation (LD0 to LD60), and the fattening phase (LD80 to LD180) [19]. Across these different periods, we used heatmap visualization to show the changes in the expression signals of all 39,640 detectable TEs (Figure 1c). In line with the previously mentioned higher number of detectable TEs before birth, we found that in prebirth skeletal muscle, the majority of TEs exhibited higher transcriptional activity. This observation points to the presence of distinct molecular regulatory circuits in the skeletal muscle at various developmental stages, with a more prominent role of TE-mediated gene regulation during the formation of primary and secondary myotubes before birth.

### 3.2. Genomic Distribution and Epigenetic Features of TE Expression

**Figure 2:**
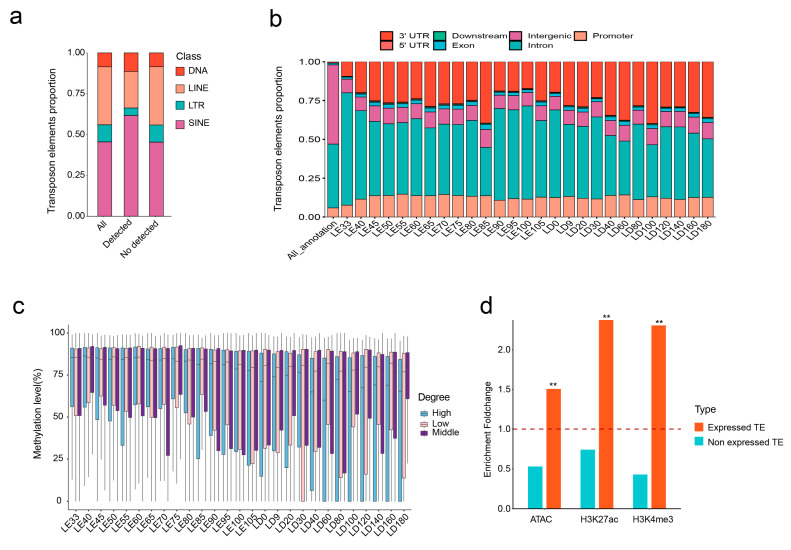
Systematic analysis of molecular characteristics of detectable TEs. (**a**) Differential distribution of transposon classes between detectable and nondetectable TEs’ expression. (**b**) Genomic distribution changes for detectable TEs at different developmental stages, compared with all TEs in the genome. (**c**) Relationship between different expression levels of TEs and their methylation levels. (**d**) Enrichment differences in epigenetic signals (ATAC, H3K27ac, H3K4me3) between expressed TEs and nonexpressed TEs, with ** indicating an adjusted *p*-value of <0.01. The dashed line represents a fold change of 1.

To gain a deeper understanding of the molecular characteristics of TE expression in porcine skeletal muscle, we carried out a comprehensive analysis of the expressed TEs. Firstly, within these expressed TEs, we identified the presence of four primary TE classes: LINE, SINE, LTR, and DNA. Notably, in comparison to the nonexpressed TEs, the expressed TEs exhibited an enrichment of SINE and DNA classes (Figure 2a). Subsequently, we investigated the distribution of TEs expressed in each developmental stage in different genomic regions. We observed that these expressed TEs were primarily enriched within gene introns, promoters, and 3′ UTR regions (Figure 2b). This suggests that TE insertions can potentially influence both the structure and expression level of genes.

Epigenetic modifications play a pivotal role in regulating TE transcriptional activity. Hence, we investigated the relationship between various epigenetic signals and TE transcription in porcine skeletal muscle. First, we explored the relationship between TE expression levels and methylation modifications. We categorized TEs into levels of high, middle, and low based on their expression at each stage. During the majority of the developmental stages, our analysis unveiled a distinct inverse correlation between TE transcriptional activity and methylation levels. Notably, this trend became more prominent following birth (Figure 2c).

In addition, we collected ATAC, H3K27ac, and H3K4me3 peak signal data from porcine skeletal muscle and compared the enrichment patterns of expressed and nonexpressed TEs in these three epigenetic signals. To our surprise, we observed a significant enrichment of expressed TEs in regions with active epigenetic signals (Figure 2d). This suggests that accessible chromatin regions may provide opportunities for the binding of transcription factors to TEs, thereby regulating their transcription. In summary, we systematically investigated various molecular features associated with expressed TEs in porcine skeletal muscle, which implicated their potential transcriptional mechanisms.

### 3.3. Self-Expressed TEs Regulate Skeletal Muscle Development

**Figure 3:**
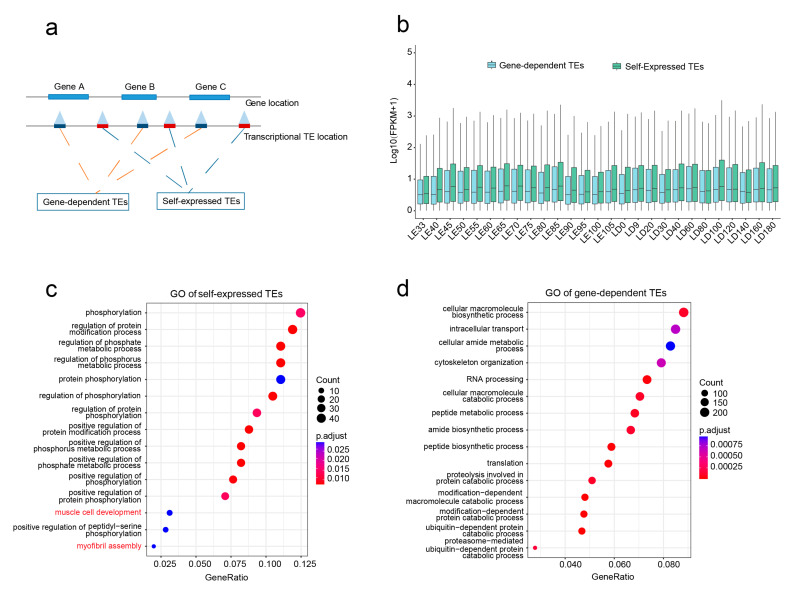
Self-expressed TEs demonstrate enhanced regulatory potential in muscle development. (**a**) Classification of self-expressed TEs and gene-dependent TEs. (**b**) Differential expression levels between self-expressed TEs and gene-dependent TEs. (**c**) Functional enrichment analysis of self-expressed TEs, the GO terms highlighted in red indicate association with skeletal muscle development. (**d**) Functional enrichment analysis of gene-dependent TEs.

The identification of a substantial number of expressed TEs located within genes poses a challenge: discerning whether a TE-mapping RNA-Seq read originates from a gene promoter or a TE promoter [52]. Following an approach taken in a previous study [53], we categorized TEs into two distinct types based on their genomic position relative to genes. Those located within genes were defined as gene-dependent TEs, while those in intergenic regions were referred to as self-expressed TEs (Figure 3a). Using this specific definition criterion, a total of 6074 self-expressed TEs were identified, accounting for 15.3% of all expressed TEs (Table S1).

An impressive discovery was that at each developmental stage, the expression levels of self-expressed TEs were consistently higher than those of gene-dependent TEs (Figure 3b). Subsequent GO analysis revealed that self-expressed TEs were enriched in biological pathways related to muscle development, such as muscle cell development and myofibril assembly pathways (Figure 3c). In contrast, gene-dependent TEs primarily enriched pathways related to fundamental metabolic processes (Figure 3d). This suggests that self-expressed TEs maintain relatively high transcriptional activity throughout skeletal muscle development, potentially exerting strong regulatory control over muscle development and maintenance. Therefore, further study of self-expressed TEs might provide a unique perspective to unravel the molecular mechanisms underlying porcine skeletal muscle development.

### 3.4. WGCNA Identification of the Key Modules Regulating Muscle Development

**Figure 4:**
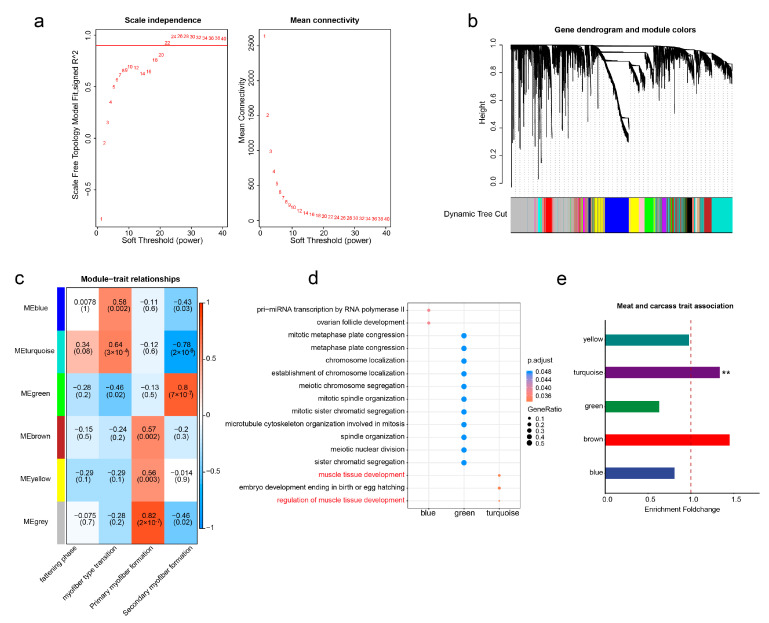
Identification of key TE modules impacting muscle development through WGCNA analysis. (**a**) Scale-free topology model fit and gene mean connectivity under different soft threshold powers. The fit index curve indicates that a soft threshold power above 22 provides scale-free topology above 0.9. (**b**) Clustering dendrogram of genes and module division by WGCNA. (**c**) Correlation analysis between the modules after merging and the characteristics of skeletal muscle at different developmental stages. (**d**) Bubble chart displaying GO enrichment results for nearby genes of TEs within different modules. (**e**) Enrichment levels of TEs within different modules on GWAS signals for pig meat and carcass traits, with ** indicating an adjusted *p*-value of <0.01. The dashed line represents a fold change of 1.

To study the interactions of self-expressed TEs in regulating skeletal muscle development, we employed a weighted gene co-expression strategy on the expression matrix (FPKM) of these TEs across the 27 examined developmental stages. As illustrated in Figure 4a, setting the soft power to 22 yielded a fitting index R2 of the scale-free network exceeding 0.9. Subsequently, we computed the co-expression relationships between TEs using the topological overlap matrix (TOM) dissimilarity and conducted module clustering (Figure 4b). After module merging, we identified six co-expression TE modules (Figure 4c). Notably, these modules show correlations with distinct stages of skeletal muscle development. Specifically, the brown, yellow, and gray modules are positively associated with primary myofiber formation, while the green module is linked to secondary myofiber formation. Additionally, the blue and turquoise modules are positively correlated with myofiber type transformation. It is vital to emphasize that the gray module is a software-defined module representing TEs with no co-expression relationships. Hence, we directed our attention towards the other five modules—blue, green, brown, yellow, and turquoise—with the aim of exploring their potential roles in the regulation of muscle development. Of these five modules, the turquoise module revealed a positive correlation with myofiber type transition (Figure 4c) and was significantly enriched in biological pathways related to the regulation of muscle tissue development (Figure 4d). This suggests the crucial regulatory role of the turquoise module in pig muscle development.

Furthermore, we investigated the genetic contributions of these modules to muscle-related traits. Specifically, we conducted an enrichment analysis of self-expressed TEs within the five modules in GWAS signals of meat and carcass traits in pigs. As expected, the results demonstrated that only TEs within the turquoise module exhibited significant enrichment in meat and carcass traits (Figure 4e), suggesting the substantial genetic impact of this module in skeletal muscle development.

To identify critical transcription factors acting on the turquoise module, we performed motif enrichment analysis on the self-expressed TEs within the module. As a control, we randomly selected an equivalent number of TEs from other self-expressed TEs to serve as a reference for the transcription factor enrichment analysis. Our results showed that the TEs within the turquoise module displayed significant and specific enrichment for well-known regulators of skeletal muscle development, including MYOD [54] and TCF12 [55] (Supplementary Figure S2). Collectively, these findings suggest the critical importance of TEs in the turquoise module, which might act as hub TEs in the regulation of skeletal muscle development.

### 3.5. Construction of TE-Mediated Gene Regulatory Networks

**Figure 5:**
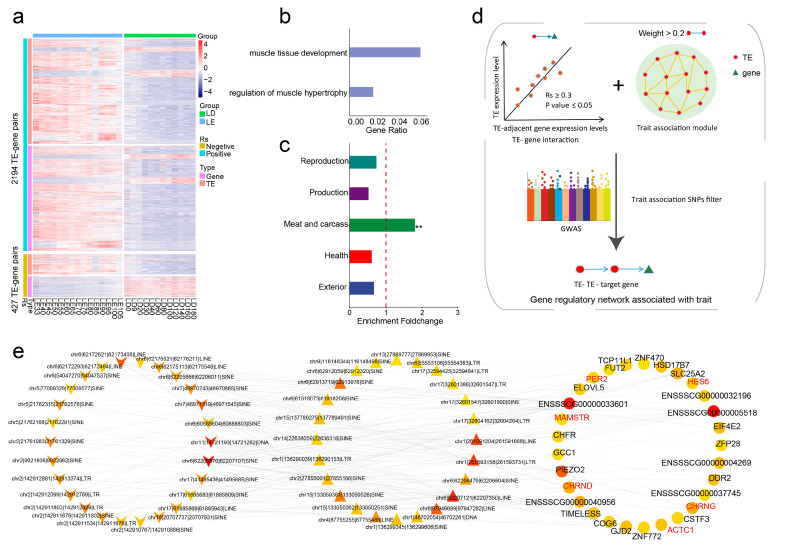
Construction of a TE-mediated gene regulatory network associated with skeletal muscle development. (**a**) Heatmap illustrating the positive or negative regulatory relationships between TEs and their target genes within the turquoise module across 27 developmental stages. (**b**) GO enrichment analysis of target genes regulated by TEs within the turquoise module. (**c**) Enrichment levels of the TE–TE–gene regulatory network on GWAS signals for five major economic traits in pigs, with ** indicating an adjusted *p*-value of <0.01. (**d**) Flowchart depicting the methodology for constructing a TE-mediated gene regulatory network. (**e**) Visualization of the TE–TE–gene regulatory network using Cytoscape software, with node importance calculated using the cytoHubba plugin. Deeper colors indicate higher connectivity, while lighter colors indicate lower connectivity.

The genetic regulatory mechanisms underlying complex traits or diseases often involve multilevel molecular regulations [56,57]. We already demonstrated the significant role played by TEs within the turquoise module in regulating skeletal muscle development. To further elucidate the specific genetic mechanisms through which these TEs control skeletal muscle development, starting from the TEs within the turquoise module, we adopted an innovative approach to construct a gene regulatory network mediated by these TEs (Figure 5d). This enabled us to meticulously dissect and gain a detailed understanding of the precise genetic mechanisms by which these TEs orchestrate the regulation of skeletal muscle development.

Here, we further refined TE target genes by evaluating the expression between TEs and their neighboring genes across 27 developmental stages. In the end, we identified 2194 TE–gene pairs with a positive regulatory relationship and 427 TE–gene pairs with a negative regulatory relationship (Figure 5a). Importantly, we found that these target genes were enriched in only two biological pathways: muscle tissue development and regulation of muscle hypertrophy (Figure 5b). This suggests the reliability of our approach in identifying the target genes.

WGCNA analysis is frequently used in studying gene–gene interactions. Using this approach, we also identified TE–TE pairs within the turquoise module that possess strong and robust interactive capabilities. Subsequently, by incorporating TE–gene pairs, we established an initial TE–TE–gene regulatory network that elucidates interactions among TEs and reveals the mechanisms through which TEs exert their influence on phenotypes via gene regulation. By comparing it with the GWAS signals of five major economical traits in pigs, we found that the TE–TE–gene regulatory network exhibited specific enrichment in meat and carcass traits (Figure 5c), thereby further confirming its substantial contribution to skeletal muscle development and growth.

Lastly, we refined the hub TE–TE-gene network by incorporating meat and carcass GWAS signals. Briefly, a TE was considered as a hub if it had SNPs that were within ±20 kb of meat- and carcass-associated SNPs (Figure 5d). The rationale is that a trait-associated SNP might affect the cis-regulatory activity of the TE where it is located, thereby affecting the expression level of critical genes involved in skeletal muscle development and growth. Within this finalized hub TE–TE–gene network (Table S2), we discovered numerous target genes that are intricately linked to muscle development and maintenance, such as HES6 [58], CHRNG [59], ACTC1 [60], CHRND [61], MAMSTR [62], and PER2 [63] (Figure 5e). Subsequently, we subjected these target gene sets to enrichment analysis on human phenotypes, revealing significant enrichment in phenotypes that are closely associated with muscle development, including multiple pterygia, aplasia of the musculature, and amyoplasia (Supplementary Figure S3). Furthermore, there were also many novel genes that have not been explicitly reported. Follow-up studies of these genes might broaden our understanding of the regulatory mechanisms underlying skeletal muscle development and growth.

In summary, our work not only included the construction of a TE–TE–gene network that sheds light on the cis- and trans-regulatory factors implicated in skeletal muscle, but also presents a novel approach to prioritizing critical TEs and genes underlying the formation of complex economic traits.

## 4. Discussion

The pig, a significant domesticated animal, represents a rich source of protein for human consumption through pork. To enhance the quality and yield of pork through genetic improvement, previous research has leveraged diverse multi-omics strategies to deepen our understanding of the genetic regulatory mechanisms underlying skeletal muscle development [16,20,22]. Nonetheless, a crucial regulatory element—TEs, constituting approximately 43.4% of the pig genome—has often been overlooked [64]. In this study, we established the first dynamic expression atlas of TEs encompassing the entire spectrum of pig skeletal muscle development. We systematically unraveled both the molecular characteristics and the functional significance of TE expression within muscle tissue. Additionally, we developed an innovative analytical framework to construct a comprehensive TE–TE–gene transcriptional regulatory network that is highly pertinent to skeletal muscle development. Our approach not only facilitates the prioritization of genes that are crucial to skeletal muscle development but also provides a general methodology to identify potential causal genes of farm animal economic traits from a TE-centric perspective.

The quality of our TE expression atlas is supported by several observations and by previous research. For example, within the expressed TEs that we identified, the proportion of gene-dependent TEs was significantly higher than that of self-expressed TEs. However, self-expressed TEs demonstrated a stronger potential for regulating muscle tissue development. These findings align with Chang’s discoveries in zebrafish, where it was found that approximately two-thirds of TE transcripts might be driven by nearby gene promoters, and self-expressed TEs exhibited tightly regulated expression patterns during zebrafish embryonic development [53].

Additionally, we uncovered a negative correlation between TE methylation levels and their expression, aligning with prior research indicating that DNA methylation can inhibit transposon expression and mobility [65,66,67]. Moreover, our study revealed a notable enrichment of transcribed TEs within regions exhibiting chromatin accessibility and active histone modifications. This finding is consistent with an earlier report suggesting that TE expression is regulated through chromatin modifications and interactions with transcription factors [68].

A noteworthy highlight of this study is our innovative approach to constructing gene regulatory networks. We chose a TE-centric approach due to the significantly higher transcription signals of self-expressed TEs observed in muscle tissue and the notable enrichment of these TEs in functions that are relevant to muscle biology. As the genetic regulation of complex traits involves a multilevel molecular process, we extended the network by first applying WGCNA to construct an initial TE–TE–gene regulatory network. This network encompasses regulatory modules that are highly relevant to skeletal muscle development and enriched in GWAS signals of meat and carcass traits. Subsequently, the hub components of the network were identified by overlapping TEs containing SNPs that are highly linked to the GWAS signals. The rationale for this approach is to utilize naturally occurring DNA variants as a form of perturbation experiment, establishing the connection between cis-regulatory elements and phenotypes. Taken together, this integration of population genetics data and functional genomics data greatly enhances our ability to leverage TEs in unraveling the genetic mechanisms that underlie muscle-related traits.

In the TE–TE–gene network, we pinpointed numerous genes that have previously demonstrated close associations with muscle-related traits. Notable examples include HES6, a known regulator of myogenic differentiation [58,69]; CHRNG and CHRND, linked to conditions like multiple pterygia and myasthenia gravis [59,61]; Mamstr, implicated in the regeneration of damaged muscle tissue [62]; and PER2, involved in the circadian rhythm of myogenic differentiation [63]. The discovery of these genes not only validates our method of constructing the regulatory network but also underscores the potential significance of previously underexplored genes in muscle development research. With future experimental validation, there is promising potential for some of these newly identified genes to act as pivotal candidate targets for improving pork production performance.

One limitation of our study is the reliance on expression correlations to construct targeted regulatory relationships within the regulatory network. Previous studies have reported that the neighboring genes of TEs are their potential target genes [36,70], and we identified TE target genes by examining the coordinated changes in TE and neighboring gene expression levels across the 27 developmental stages. While this method can be accurate when samples spanning a large number of developmental stages are available, it may be less suitable in scenarios with limited sampling. However, as multi-omics data continue to advance, we can enhance our approach by integrating Hi-C technology to precisely capture genomic interaction information at each individual stage [71]. This would allow us to determine stage-specific gene regulatory networks. Additionally, advancements in single-cell sequencing techniques and the tools for quantifying TEs at the single-cell level would allow for a finer resolution for the TE–TE–gene regulatory network [35,36]. This advancement promises a more comprehensive understanding of the genetic regulatory mechanisms underpinning complex traits or diseases.

## 5. Conclusions

To our knowledge, this is the first ever establishment of a dynamic expression landscape of TEs during the development of pig skeletal muscles. It unveiled the distribution of transcriptionally active TEs within the genome and their associations with epigenetic modifications. Furthermore, we pioneered the innovative construction of a gene regulatory network centered on TEs, identifying potential genes that are closely implicated in the regulation of skeletal muscle development. In summary, our research not only provides vital candidate targets for enhancing pork production traits but also offers a general method for dissecting the genetic basis of complex traits.

## Data Availability

Data are contained within the article and Supplementary Materials.

## Supplementary Materials

The following supporting information can be downloaded at: https://www.mdpi.com/article/10.3390/ani13223581/s1. Figure S1: GO enrichment analysis of genes proximate to TEs with specific expression during the LE33 developmental stage; Figure S2: Bubble plot illustrating the differences in transcription factor motif enrichment between TEs within the turquoise module and an equal number of self-expressed TEs outside the module; Figure S3: Enrichment analysis of the target gene set on human phenotypes; Table S1: The expression matrix of self-expressed TEs across 27 developmental time points; Table S2: The refined hub TE–TE–gene regulatory network post GWAS signal filtering.
