# Supplementary material for: An Analysis of a Transposable Element Expression Atlas during 27 Developmental Stages in Porcine Skeletal Muscle: Unveiling Molecular Insights into Pork Production Traits
Source: Animals (Basel). 2023 Nov 20;13(22):3581. doi: 10.3390/ani13223581 (PMC10668843; doi:10.3390/ani13223581)
Supplement: Supplementary file 1 [file animals-13-03581-s001.zip › Supplementary-Figure.pdf]

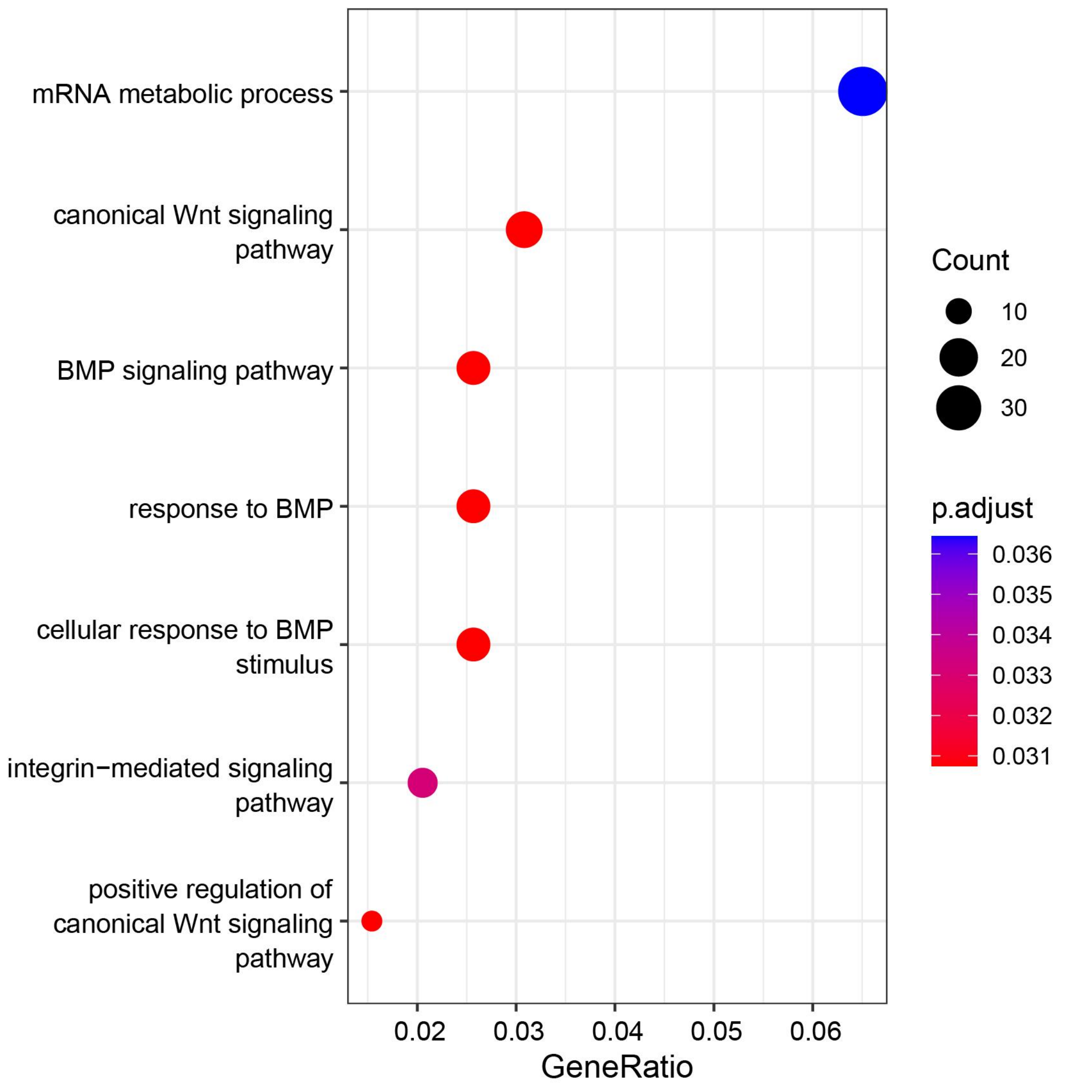

Figure S1. GO enrichment analysis for genes proximate to TEs with specific expression during the LE33 developmental stage.

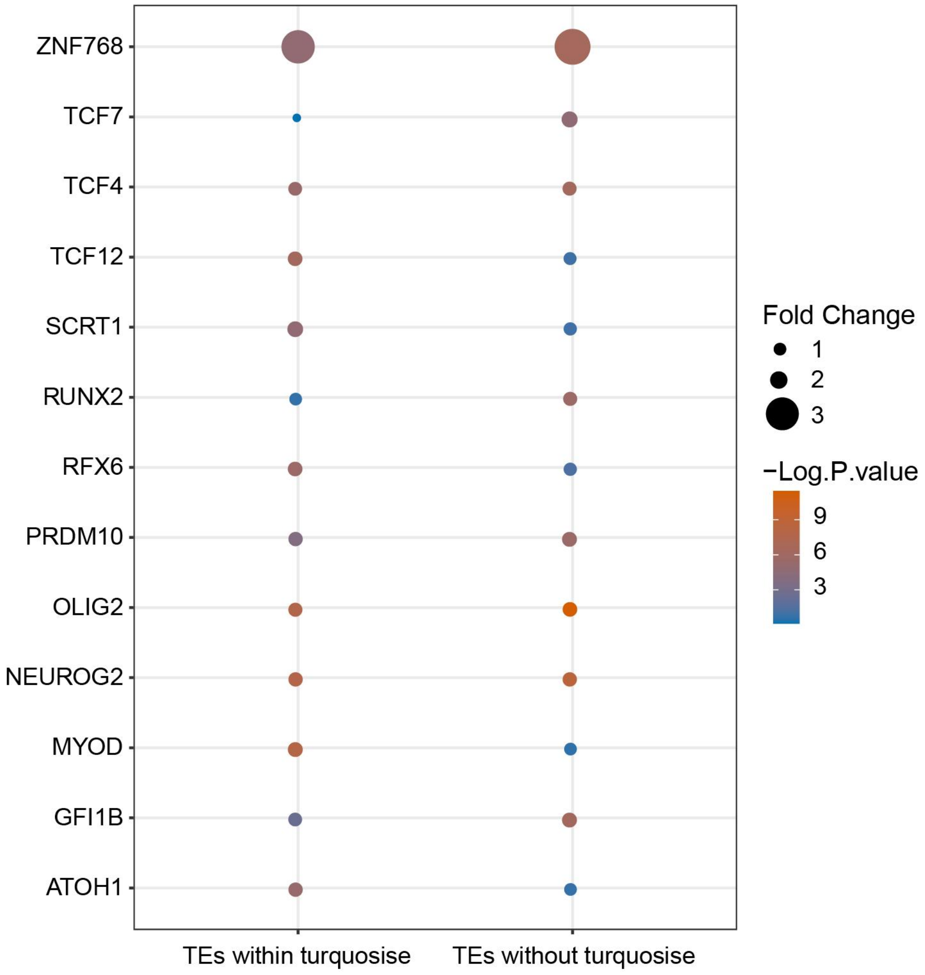

Figure S2. Bubble plot illustrating the differences in transcription factor motif enrichment between TEs within the turquoise module and an equal number of self-expressed TEs outside the module.

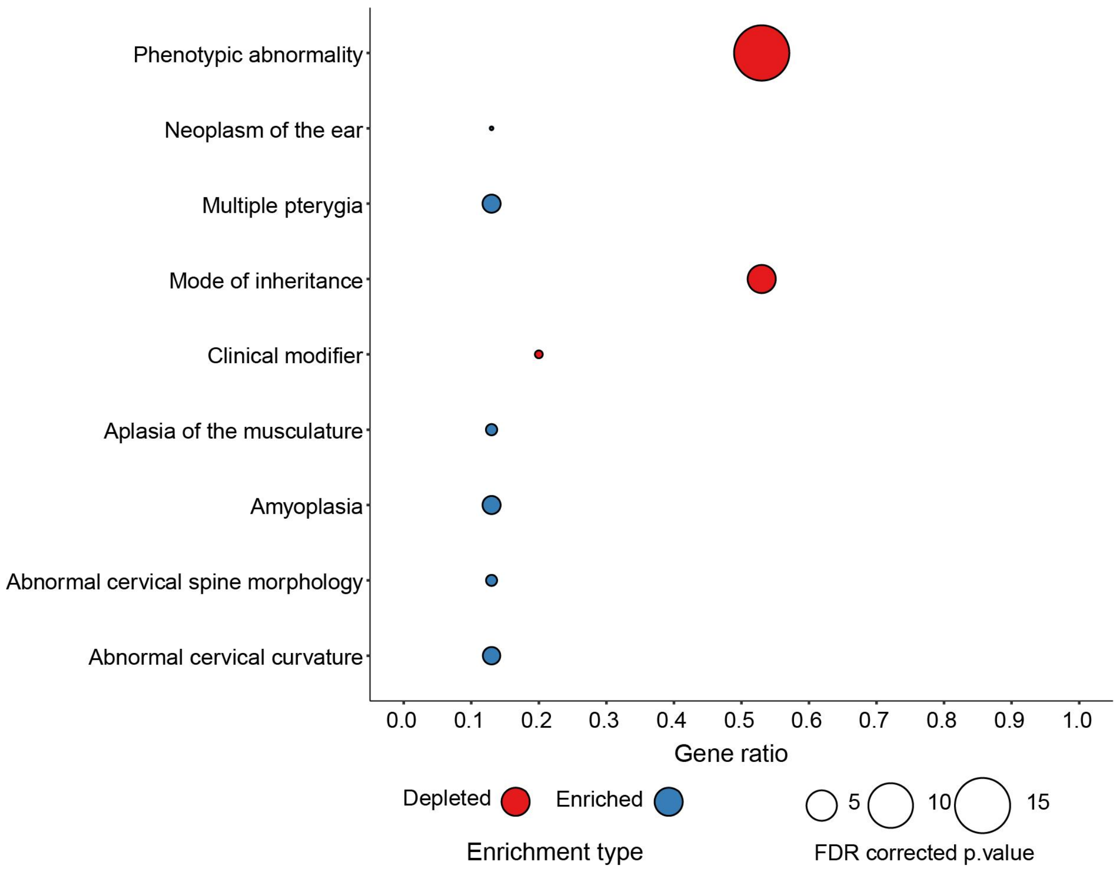

Figure S3. Enrichment analysis of target gene set on human phenotypes.
